# Stratification of cardiovascular risk in patients with atrial fibrillation and obstructive sleep apnea—validity of the 2MACE score

**DOI:** 10.1007/s11325-017-1469-6

**Published:** 2017-02-02

**Authors:** Anna E. Platek, Filip M. Szymanski, Krzysztof J. Filipiak, Alicja Dudzik-Plocica, Bartosz Krzowski, Grzegorz Karpinski

**Affiliations:** 1st Department of Cardiology, Medical University of Warsaw, 1A Banacha Street, 02-097 Warsaw, Poland

**Keywords:** Obstructive sleep apnea, Cardiovascular risk, Risk assessment

## Abstract

**Background:**

Risk stratification in patients with atrial fibrillation (AF) is critically important because this group is at high risk of mortality and morbidity. One of the comorbidities potentially affecting thromboembolic and total cardiovascular risk is obstructive sleep apnea (OSA). The aim of this study was to determine whether or not patients with atrial fibrillation and concomitant obstructive sleep apnea have a higher predicted cardiovascular risk than those without sleep-disordered breathing.

**Methods:**

The study was designed to be a cross-sectional observational study. Consecutive patients with primary diagnosis of AF who qualified for first-ever catheter ablation between 2011 and 2013 were enrolled. All patients had an overnight polysomnography performed for the diagnosis of OSA and calculation of a 2MACE score—a cardiovascular risk assessment score for AF.

**Results:**

We studied 211 AF patients (mean age 57.1 ± 10.2 years, 62.6% males). OSA with apnea-hypopnea index (AHI) ≥15/h was found in 48 patients (22.7%). Cardiovascular disease and risk factors were as follows: 8 (3.8%) patients had congestive heart failure, 27 (12.8%) diabetes, 16 (7.6%) history of stroke or thromboembolic disease, 194 (91.9%) arterial hypertension, 24 (11.4%) vascular disease, and 31 (14.7%) were current smokers. A significantly higher percentage of patients with OSA was at high risk of cardiovascular disease (29.2 vs. 8.1%; *p* < 0.0001). The trend remained significant in different categories of obstructive sleep apnea when categorized by AHI into non-OSA, and mild, moderate, and severe OSA. Similarly, the mean 2MACE score was statistically significantly higher in OSA than non-OSA patients (2.1 ± 1.1 vs. 1.4 ± 1.0; *p* < 0.0001).

**Conclusion:**

OSA prevalence is increased in AF patients and is associated with an increase 2MACE score—an indicator of major cardiovascular events. There is a linear relationship between severity of OSA and increasing 2MACE scores, indicating increasing cardiovascular risk related to OSA severity.

## Introduction

Patients with atrial fibrillation (AF) are at increased risk of cardiovascular disease (CVD) and death [1]. Therefore, extensive measures are applied in order to stratify their risk (i.e., of bleeding and thromboembolism). To effectively manage the risk in patients with AF, it is useful to use assessment scales such as CHA_2_DS_2_-VASc, HAS-BLED, or the 2MACE score [2, 3] which was recently developed for assessment of cardiovascular risk in AF patients.

Obstructive sleep apnea (OSA) is a disease highly prevalent in patients with AF. It is also a strong risk factor for CVD in the general population. Unfortunately, OSA is not routinely included in any of the risk assessment schemes for AF. Currently, there are no data on how the presence of OSA affects risk assessment and if OSA impairs the validity of risk assessment scores.

The aim of this study was to determine whether or not patients with AF and concomitant OSA have a higher predicted cardiovascular risk than those without OSA, according to the 2MACE score.

## Methods

All study design details were described previously [4]. The study was designed to be a cross-sectional observational study. Consecutive patients with primary diagnosis of AF who qualified for first-ever catheter ablation between 2011 and 2013 were enrolled. Inclusion criteria were age ≥18 years, confirmed diagnosis of nonvalvular AF, and prequalification for invasive treatment of arrhythmia (catheter ablation or cardioversion). Patients with valvular AF, myocardial infarction, or decompensation of heart failure within 6 months prior to study entry, estimated life expectancy of ≤6 months, acute and/or chronic pulmonary diseases such as obstructive pulmonary disease or active tuberculosis, neuromuscular disease, hemochromatosis, severe neurologic or psychiatric disorders, or patients who did not give an informed consent were excluded from the study. The study was designed and conducted with the accordance of the Declaration of Helsinki and was approved by the University Ethics Committee.

### Diagnosis of atrial fibrillation

Diagnosis of AF was made in accordance with the European Society of Cardiology Guidelines, requiring at least one arrhythmia episode recorded in a 24-h ECG Holter monitor during the 6 months before study enrollment. Paroxysmal AF was defined as self-terminating (up to 7 days), while persistent AF was diagnosed when an arrhythmia episode lasted longer than 7 days or required termination by cardioversion.

### Cardiovascular risk assessment

All definitions of the primary endpoints and diagnosis of concomitant disease were made according to the 2MACE validation study [3]. In the validation study, MACE included fatal/nonfatal myocardial infarction (MI), cardiac revascularization (stent or coronary artery bypass surgery/CABG), and cardiovascular death. The diagnosis of MI was made according to the definition proposed by the Joint ESC/ACCF/AHA/WHF Task Force. If a patient died within 4 weeks of MI, this event was recorded as fatal MI. Death was classified as vascular unless an unequivocal noncardiovascular cause of death was identified. Cardiovascular death included sudden death, progressive congestive heart failure, or procedure-related death (surgical or percutaneous revascularization).

Cardiovascular risk factors were also defined as in the validation study [3]: arterial hypertension—repeatedly elevated blood pressure (>140/90 mmHg) or taking antihypertensive drugs; diabetes—a random plasma glucose >200 mg/dl (11.1 mmol/l), or fasting plasma glucose >126 mg/dl (7.0 mmol/l), or 2-h plasma glucose >200 mg/dl (11.1 mmol/l) during an OGTT or taking antidiabetic drugs; heart failure—the presence of signs and symptoms typical of heart failure or reduced ejection fraction (<40%). The metabolic syndrome was defined according to modified ATP-III criteria.

The 2MACE score was calculated as follows: 2 points each were assigned for metabolic syndrome and age ≥75 years, and 1 point each for myocardial infarction/revascularization, congestive heart failure (ejection fraction <40%) and thromboembolism (stroke/transient ischemic attack) for a range of 0 to 7 points (Table 1). Patients with 2MACE score ≥3 points were considered at high cardiovascular risk.

**Table 1:** Definition of the 2MACE score

Acronym	Definition	Points
2M	History of myocardial infarction/cardiac revascularization	1
	Metabolic syndrome	2
A	Age (>75 years)	2
C	Congestive heart failure (ejection fraction <40%)	1
E	Thromboembolism	1

### Sleep study evaluation

All patients included in the study were studied with overnight polysomnography irrespective of their daytime and nighttime symptoms. Polysomnography was performed using devices recording >4 channels including channels to detect respiratory movements or respiratory effort, airflow, heart rate, ECG, and oxygen saturation (Embletta Gold; Flaga, Reykjavik, Iceland). All sleep study results were scored manually according to the current guidelines. Apnea was defined as a reduction of airflow of ≥90% of pre-event baseline lasting ≥10 s. Hypopnea was defined as a ≥30% drop in maximal airflow lasting ≥10 s, associated with ≥3% oxygen desaturation from pre-event baseline [5]. OSA was diagnosed based on the apnea-hypopnea index (AHI, the number of apneas and hypopneas per hour) and categorized into three severity classes: mild OSA–AHI ≥5 and <15 per hour, moderate OSA–AHI ≥15 and <30 per hour, and severe OSA–AHI ≥30 per hour.

### Statistical analysis

Data were tested for normality using the Kolmogorov–Smirnov test. Continuous data are presented as mean and 95% confidence intervals (CIs), with statistical comparisons performed with the Mann–Whitney test or Student’s *t* test. For categorical variables, comparison was made using either the chi-squared or Fisher exact tests. A Pearson correlation was used to determine the correlation coefficient between sleep disorder severity and 2MACE score. A one-way analysis of covariance (ANCOVA) was used to determine associations between body mass index and OSA while controlling for relevant cofactors. A *p* value of less than 0.05 was considered statistically significant. Statistical analyses were performed using SPSS (SPSS version 21, Inc., Chicago, IL).

## Results

We enrolled 211 patients with AF (mean age 57.1 ± 10.2 years, 62.6% males). The majority of patients (148, 70%) presented with paroxysmal AF, while the remaining patients had sustained AF. Prevalence of cardiovascular disease and risk factors was as follows: 8 patients (3.8%) had congestive heart failure, 27 (12.8%) diabetes, 16 (7.6%) history of stroke or thromboembolic disease, 194 (91.9%) arterial hypertension, 24 (11.4%) vascular disease, and 31 (14.7%) were current smokers or had history of smoking in two previous years. Mild OSA was found in 39 (18.5%) patients, moderate in 30 (14.2%), and severe in 18 (8.5%) patients (Table 2).

**Table 2:** Baseline characteristics of the study population

Parameter	Value
Age (years)	57.1 ± 10.2
BMI (kg/m^2^)	29.7 ± 4.8
AHI (per hour)	3.6 (1.2–13.1)
Oxygen desaturation index	4.1 ± 3.6
Mean oxygen saturation	93.4 ± 2.8
Minimal recorded saturation	91.3 ± 4.7
Female sex	79 (37.4%)
Congestive heart failure	8 (3.8%)
Diabetes mellitus	27 (12.8%)
Stroke/thromboembolism	16 (7.6%)
Arterial hypertension	194 (91.9%)
Vascular disease	24 (11.4%)
Smoking	31 (14.7%)
Metabolic syndrome	150 (71.1%)
Paroxysmal AF	148 (70.1%)
Mean 2MACE	1.7 ± 1.1
Patients with 2MACE ≥3	36 (17.1%)

Values are presented as mean ± standard deviation (SD), median (interquartile range), or *n* (%)

*AF* atrial fibrillation, *AHI* apnea-hypopnea index, *BMI* body mass index

We observed that the presence of OSA significantly influenced the prevalence of some cardiovascular diseases and risk factors, including arterial hypertension. Detailed characteristics of patients according to the presence of sleep disordered breathing severity are presented in Table 3. There were no differences regarding age, but other parameters, including sex and body mass index, were different between the groups with and without OSA.

**Table 3:** Clinical characteristics of patients according to the severity of obstructive sleep apnea (AHI)

Parameter	Non-OSA/mild OSA (*n* = 163)	Moderate to severe OSA (*n* = 48)	*p*
Age (years)	56.5 ± 10.8	59.1 ± 7.5	0.26
BMI (kg/m^2^)	29.0 ± 4.6	32.0 ± 4.8	<0.0001
AHI (per hour)	2.5 (0.9–4.7)	23.6 (19.5–33.5)	<0.0001
Oxygen desaturation index	3.1 ± 1.3	27.6 ± 3.5	<0.0001
Mean oxygen saturation	97.4 ± 1.8	91.3 ± 4.7	<0.0001
Minimal recorded saturation	93.1 ± 2.4	87.2 ± 3.6	<0.0001
Female sex	54 (33.1%)	25 (52.1%)	0.01
Congestive heart failure	1 (0.6%)	7 (14.6%)	<0.0001
Diabetes mellitus	11 (6.7%)	16 (33.3%)	<0.0001
Stroke/thromboembolism	10 (6.1%)	6 (12.5%)	0.13
Arterial hypertension	147 (90.2%)	47 (97.9%)	0.067
Vascular disease	13 (8.0%)	11 (22.9%)	0.007
Smoking	22 (13.5%)	9 (18.8%)	0.25
Metabolic syndrome	(62.1%)	(83.9%)	<0.0001
Paroxysmal AF	121 (74.2%)	27 (56.3%)	0.02
Mean 2MACE	1.4 ± 1.0	2.1 ± 1.1	<0.0001
Patients with 2MACE ≥3	13 (8.1%)	14 (29.2%)	<0.0001

Values are presented as mean ± standard deviation (SD), or median (interquartile range), or *n* (%)

*AF* atrial fibrillation, *AHI* apnea-hypopnea index, *BMI* body mass index

**Fig. 1:**
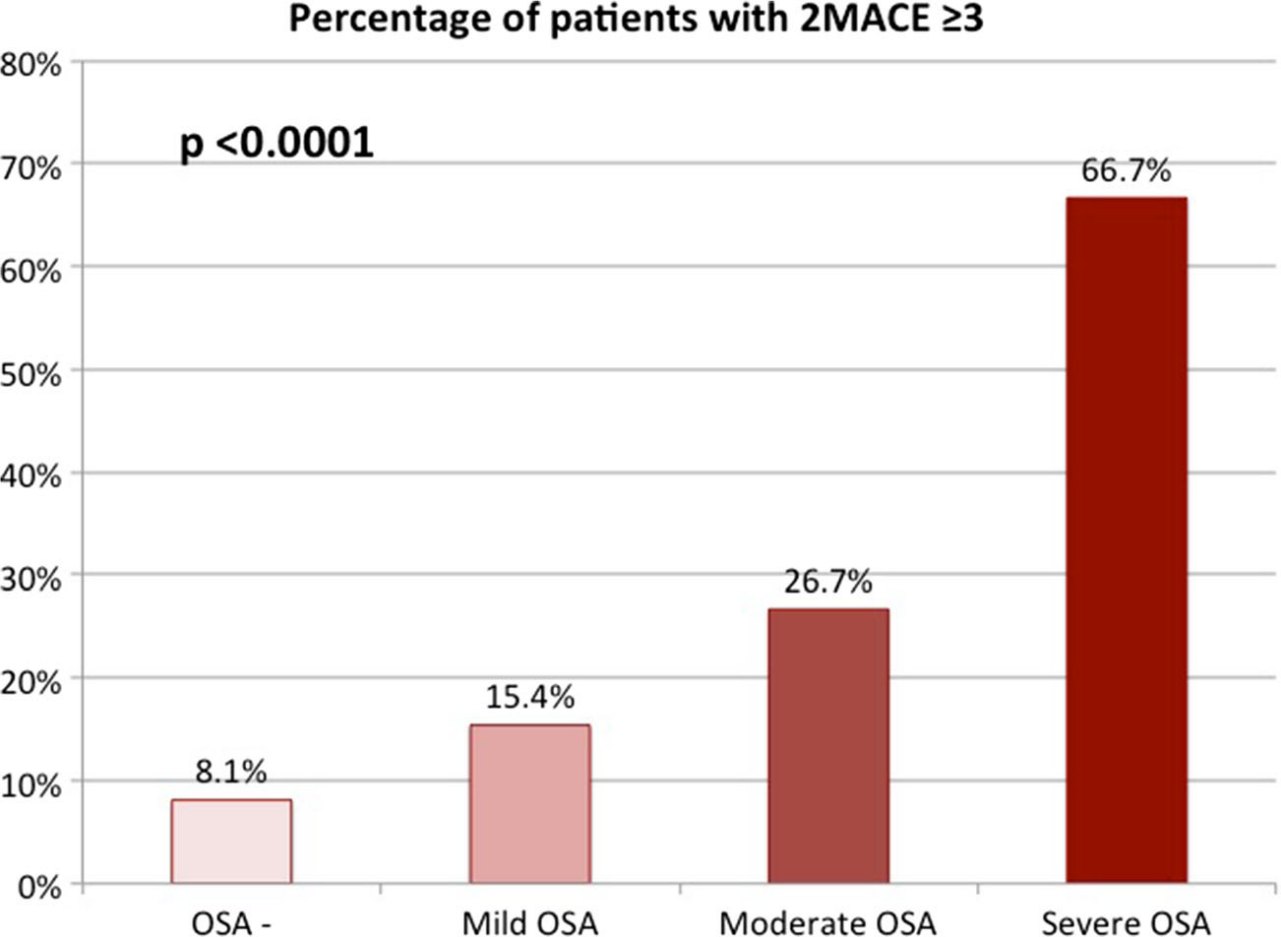
Percentage of patients with high cardiovascular risk in the 2MACE score according to the severity of obstructive sleep apnea

**Fig. 2:**
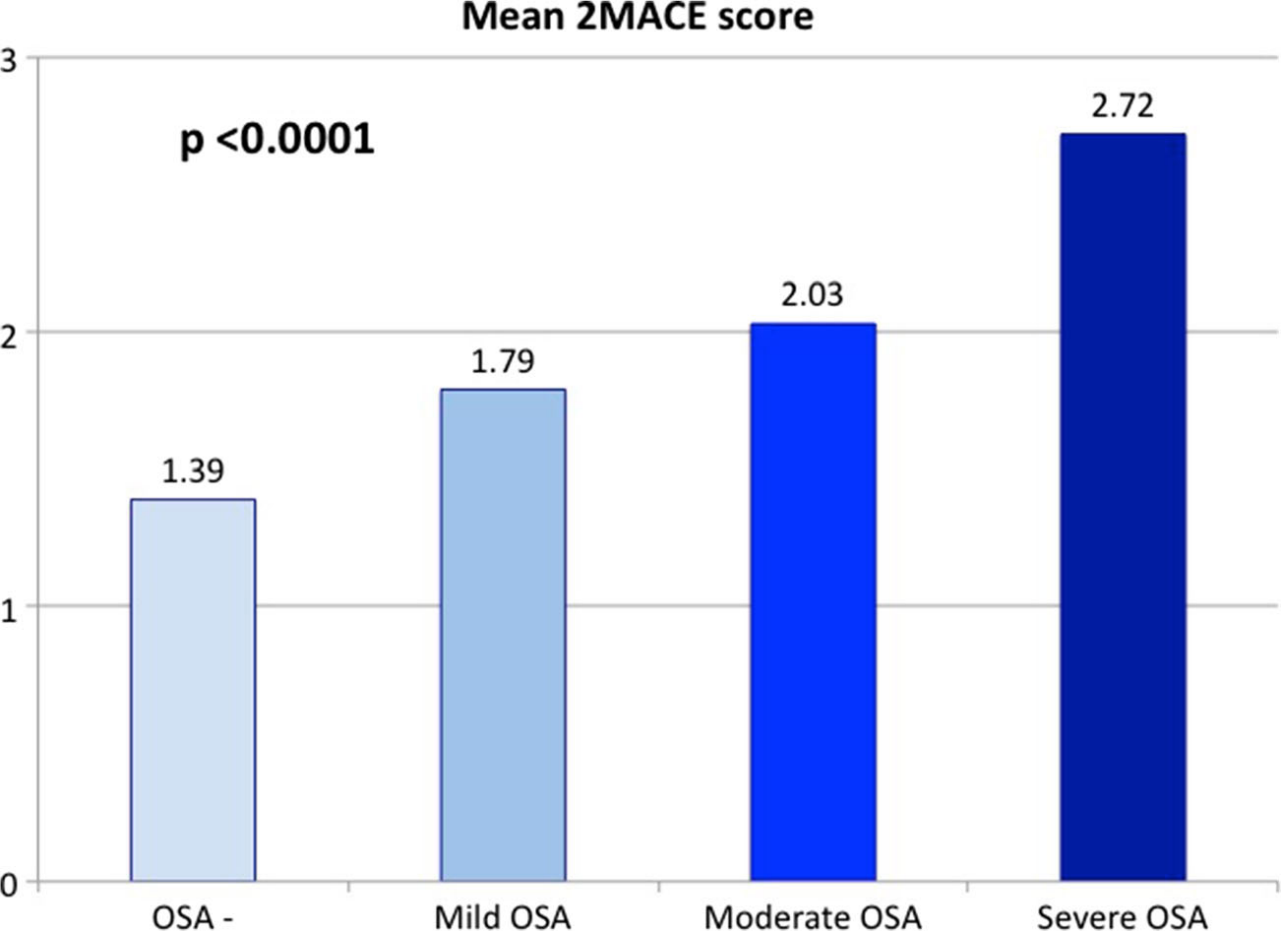
Mean 2MACE score according to the severity of obstructive sleep apnea

A significantly higher percentage of patients with OSA was at high risk for cardiovascular disease (29.2 vs. 8.1%; *p* < 0.0001). The trend remained significant in different categories of mild, moderate, and severe OSA (Fig. 1). Similar observations were made for mean 2MACE scores which were statistically significantly higher in patients with OSA than patients without OSA (2.1 ± 1.1 vs. 1.4 ± 1.0; *p* < 0.0001). The results remained significant according to severity of disease (Fig. 2). There was a weak positive correlation between AHI and 2 MACE scores (correlation coefficient = 0.369, *p* < 0.0001).

**Fig. 3:**
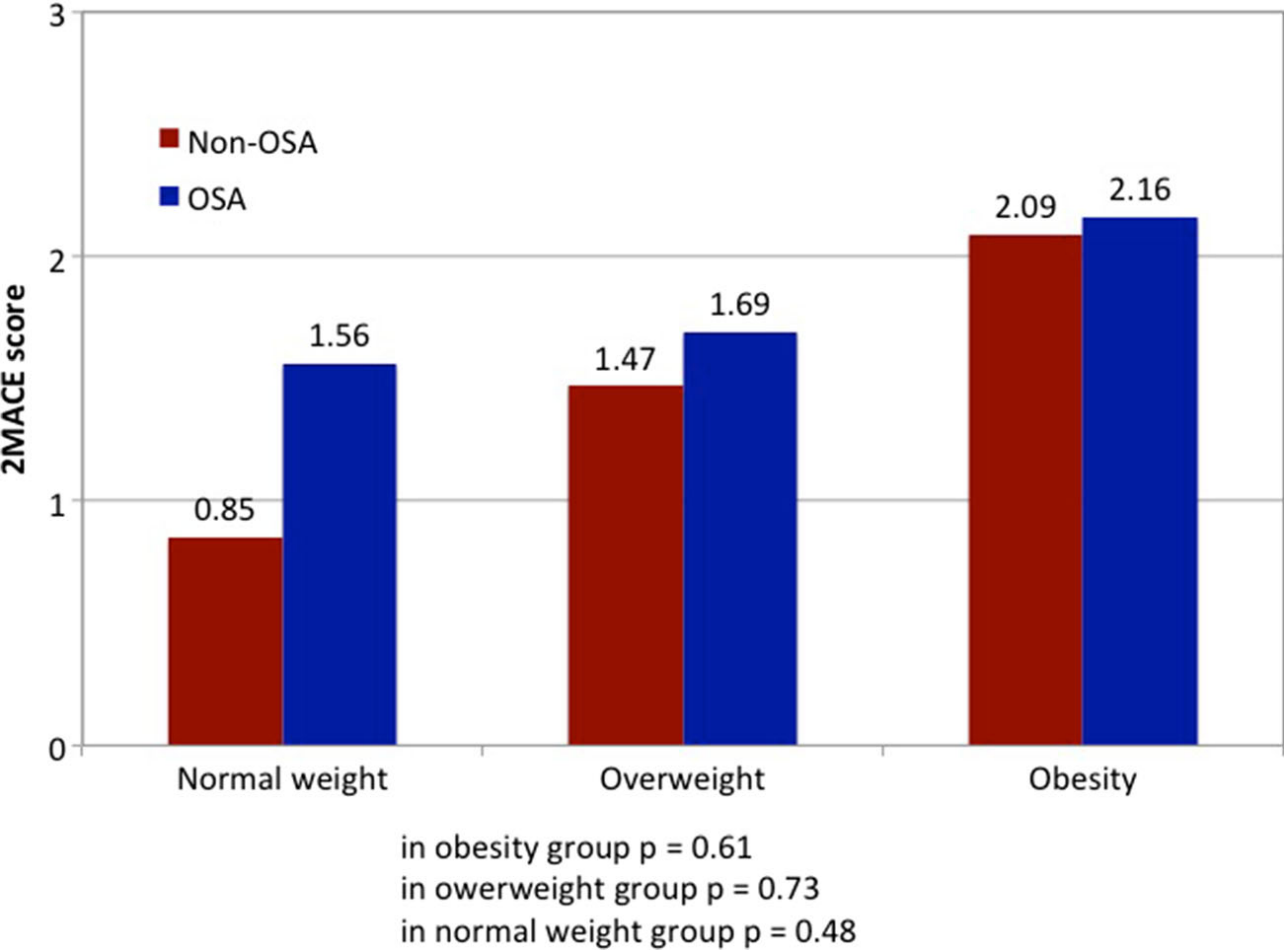
Relationship between 2MACE score and OSA index in patients with different body mass index

In order to test the association between OSA severity, 2MACE scores, and BMI, statistical analysis using ANCOVA was performed. There were 33 patients with normal body weight (BMI 18–24.99 kg/m^2^), 90 patients who were overweight (BMI 25–29.99 kg/m^2^), and 88 who patients were obese (BMI >30 kg/m^2^) (Fig. 3). There was no statistically significant difference in the mean BMI (*F* = 0.31, *p* = 0.575) or age (*F* = 0.045, *p* = 0.831) between patients with OSA and patients with no OSA. No interaction was shown between OSA and age (*F* = 0.014, *p* = 0.907).

### Discussion

The present study showed that in patients with AF, the presence of OSA is associated with an increased 2MACE score. This translates to a higher risk of cardiovascular disease seen in patients with concomitant AF and OSA. The current study also showed that cardiovascular risk assessed with the 2MACE score is correlated with higher AHI. Therefore, severity of OSA is important and should be considered in the CVD risk stratification of AF patients.

Current epidemiological studies indicate that OSA occurs in about 24% of men and 9% of women in the general population aged between 30 and 60 years [6]. Occurrence of OSA combined with signs of daytime sleepiness in approximately 4% of men and 2% of women [6]. Nevertheless, a significant proportion of cases remain undiagnosed. Data from the American Academy of Sleep Medicine show that up to 80–90% of patients with sleep disordered breathing are currently underdiagnosed [7]. Even greater is the incidence of OSA is seen in patients with atrial fibrillation. Previous studies have shown that OSA is present in nearly 50% of patients with AF [4]. In the present cohort, prevalence of moderate to severe OSA was 23%, which is greater than that in the general population. The particular importance of OSA in the present study is its association with CVD.

Risk factors that cause OSA, including obesity, older age, hormonal disorders (acromegaly, hypothyroidism), and cigarette smoking also predispose to CVD. OSA results in intermittent hypoxia, sympathetic activation, pulmonary hypertension, and variations in systemic blood pressure. This translates to a higher incidence hypertension (often resistant to therapy), coronary heart disease, myocardial infarction, heart failure, arrhythmias, pulmonary hypertension, stroke, chronic kidney disease, endothelial dysfunction, insulin resistance, metabolic disorders, erectile dysfunction, increased blood clotting, stent thrombosis, and chronic inflammation in patients with OSA [8, 9].

It is recognized that second to nonadherence to therapy, OSA is the leading cause of treatment failure in patients with hypertension [10]. Oxidative stress, chronic inflammation, and hypertension caused by OSA translate into damage to the vascular endothelium and promote the development of atherosclerotic plaques. One of the most important complications of OSA is atherosclerotic disease manifesting as stroke or myocardial infarction [11, 12].

Another important issue is the impact of OSA on cardiac arrhythmias. OSA is associated with increased incidence of premature atrial beats, sinus bradycardia, sinus pauses, premature ventricular beats, and, what is crucial in the context of this study—greatly increased incidence of AF [13]. Several studies have shown that nearly half of patients with AF meet the criteria for diagnosis of at least mild OSA [14]. The occurrence of OSA in these patients results in worse outcomes. The presence of OSA makes rhythm control strategies less efficient and makes it harder to restore sinus rhythm either with cardioversion or AF ablation [15, 16]. On the other hand, OSA worsens outcomes, because patients with OSA and AF are at increased risk of stroke and venous thromboembolism as shown by higher CHADS_2_ and CHA_2_DS_2_-VASc scores in association with higher AHI [17].

The newly developed 2MACE score is a useful tool by which patients with AF may be screened for increased risk of major cardiovascular events. However, the current study has several limitations. The present study is yet a preliminary analysis, which needs to be validated in a larger cohort. For proper validation, patients should be followed for a cardiovascular event. Furthermore, interventional studies are needed to clarify the impact of OSA treatment in high-risk patients to reverse increased cardiovascular risk and related adverse outcomes. Future investigations clarifying specific OSA and AF-related pathways of cardiovascular risk may suggest targeted preventive therapies to mitigate OSA-induced mortality and morbidity in patients with AF.

## Conclusions

OSA prevalence is increased in patients with AF and is associated with an increased 2MACE score—a predictor of major cardiovascular events. There is a linear relationship between severity of OSA and increasing 2MACE score, indicating increasing cardiovascular risk related to OSA severity.

## Abbreviations

AF: Atrial fibrillation
AHI: Apnea-hypopnea index
CABG: Coronary artery bypass grafting
CI: Confidence interval
CVD: Cardiovascular diseases
MACE: Major adverse coronary events
MI: Myocardial infarction
OSA: Obstructive sleep apnea
